# Research and Remedies

**Published:** 1912-11-16

**Authors:** 


### RESEARCH AND REMEDIES.

### Concussion of the Spine.

This term was very greatly abused about twenty years ago, and was employed to cover cases the bulk of which were really nothing but traumatic neurasthenia and sometimes actual malingering. As a result of some plain speaking and writing by Page, Gould, Bennett, Golding Bird, and other surgeons, a more correct nomenclature was adopted; and "concussion of the spine" fell into such disrepute that many regard it as a myth altogether. In the *Medical Chronicle* E. M. Corner pleads for recognition of the condition as a clinical entity, though one of considerable rarity. He quotes two interesting cases, almost identical in details, in which after severe violence to the spine complete paralysis and anæsthesia of all the limbs without loss of consciousness ensued; in both cases rapid and complete recovery occurred in about half an hour, following the administration of brandy, and in both cases skiagraphy revealed a fracture of the spinal column without pressure on the cord. Further evidence is adduced to show that in cases of actual traumatic destruction of the cord at any given level there is often, during the first few hours, temporary paralysis and anæsthesia at a higher level than that corresponding to the site of injury. Soon this passes off, leaving symptoms in accordance with the actual injury to the cord. It is suggested that this temporary loss of function, three cases of which are cited, is due to concussion of the cord above the actual injury to it.

### Muscle Changes in Disease.

Drs. Jewesbury and Topley have contributed to the Proceedings of the Royal Society of Medicine a research upon the pathological changes which take place in the muscles during general diseases. In acute general diseases muscle changes are extremely slight. True fatty degeneration, the authors find, is much less common than is usually stated; but it occurs to a marked degree in diphtheritic toxæmia, in certain blood disorders, and in phosphorus poisoning. Glycogen is not found to any notable extent in the muscles except in cases of diabetes; three such cases were investigated, and glycogen was strikingly present in the muscles in all of them. Amyloid change was never found. In wasting diseases the changes found are chiefly alterations of size, shape, and staining reactions of the fibres; increase in the nuclei, with occasional production of false giant cells; and relative increase of interstitial tissue. In certain disorders associated with abnormal carbohydrate metabolism there is a great increase in the amount of interstitial fat present; this bears no relation to true fatty degeneration, and it is doubtful whether it has any pathological significance.

